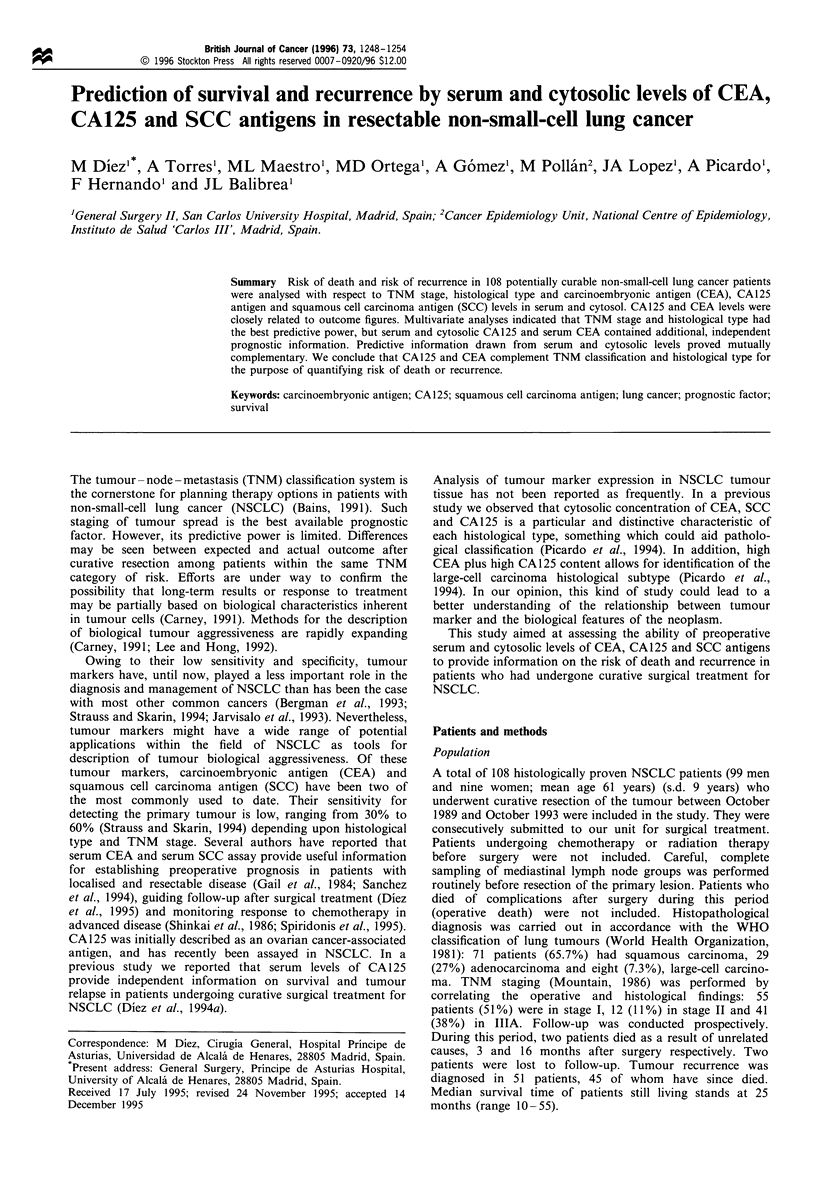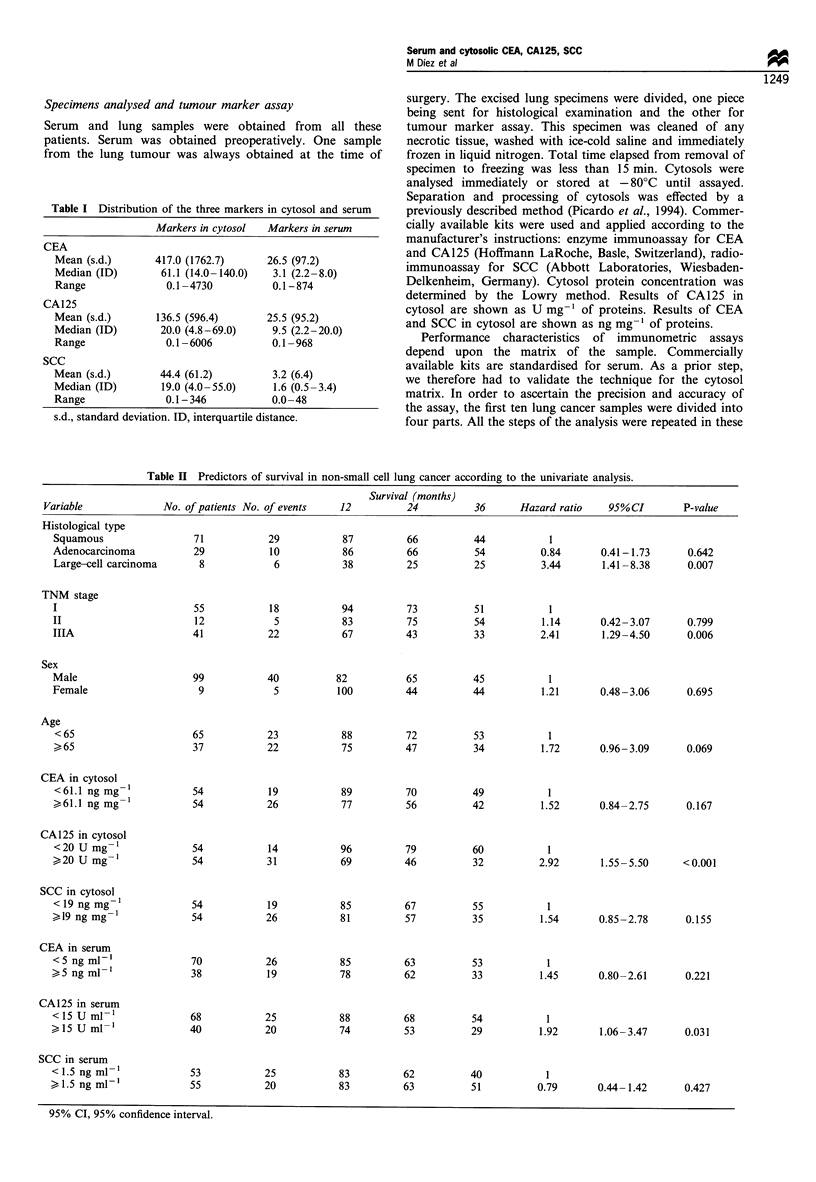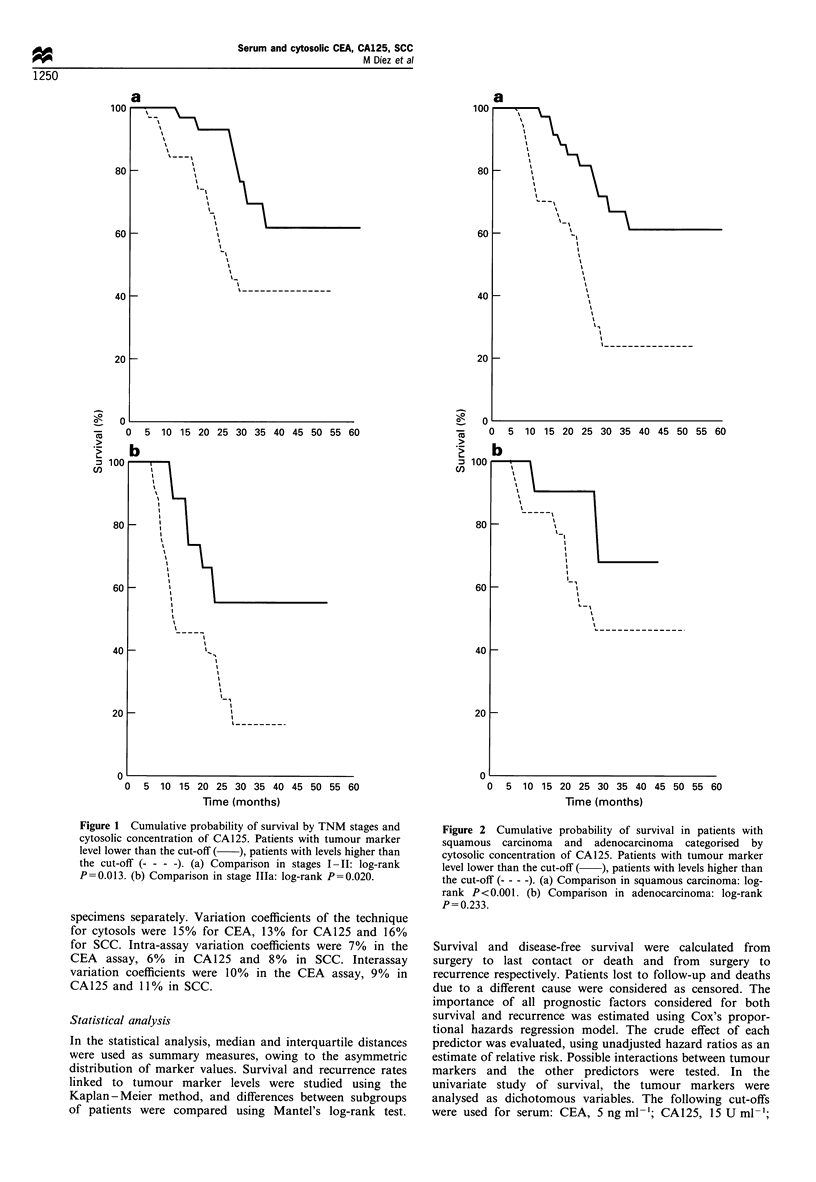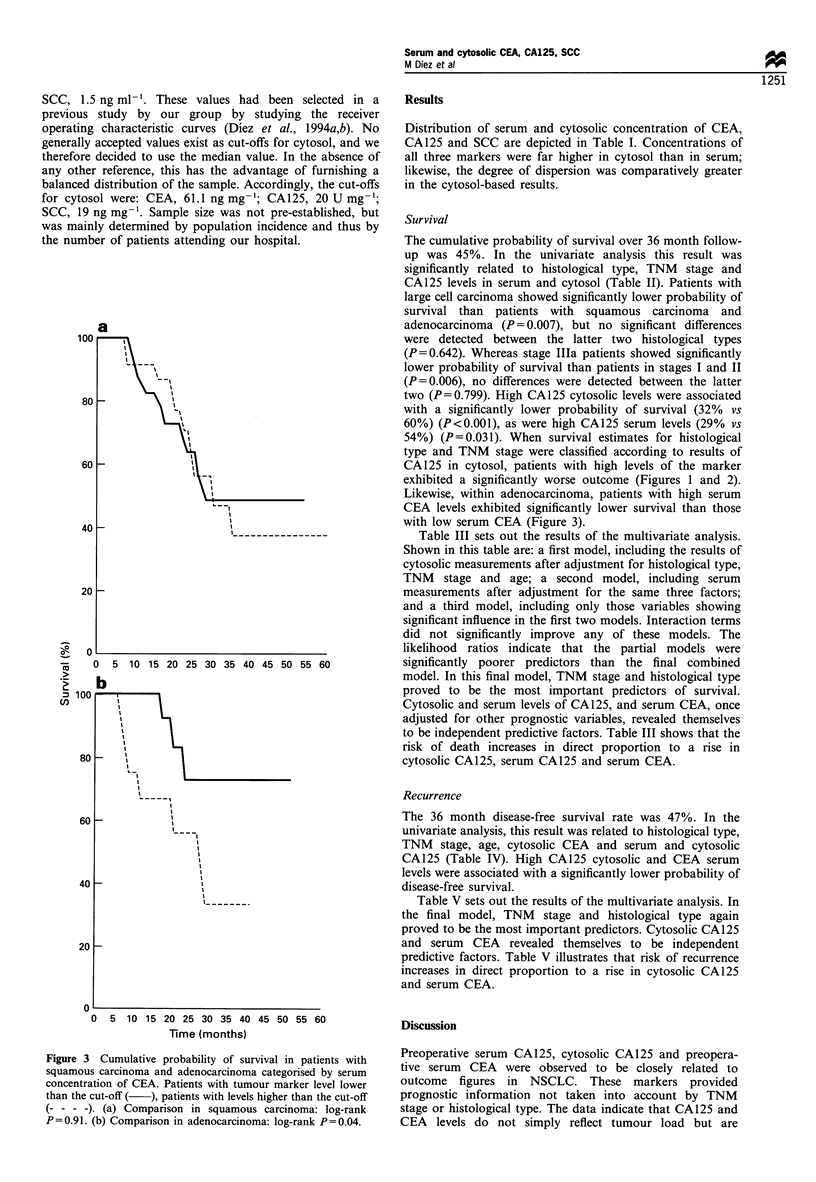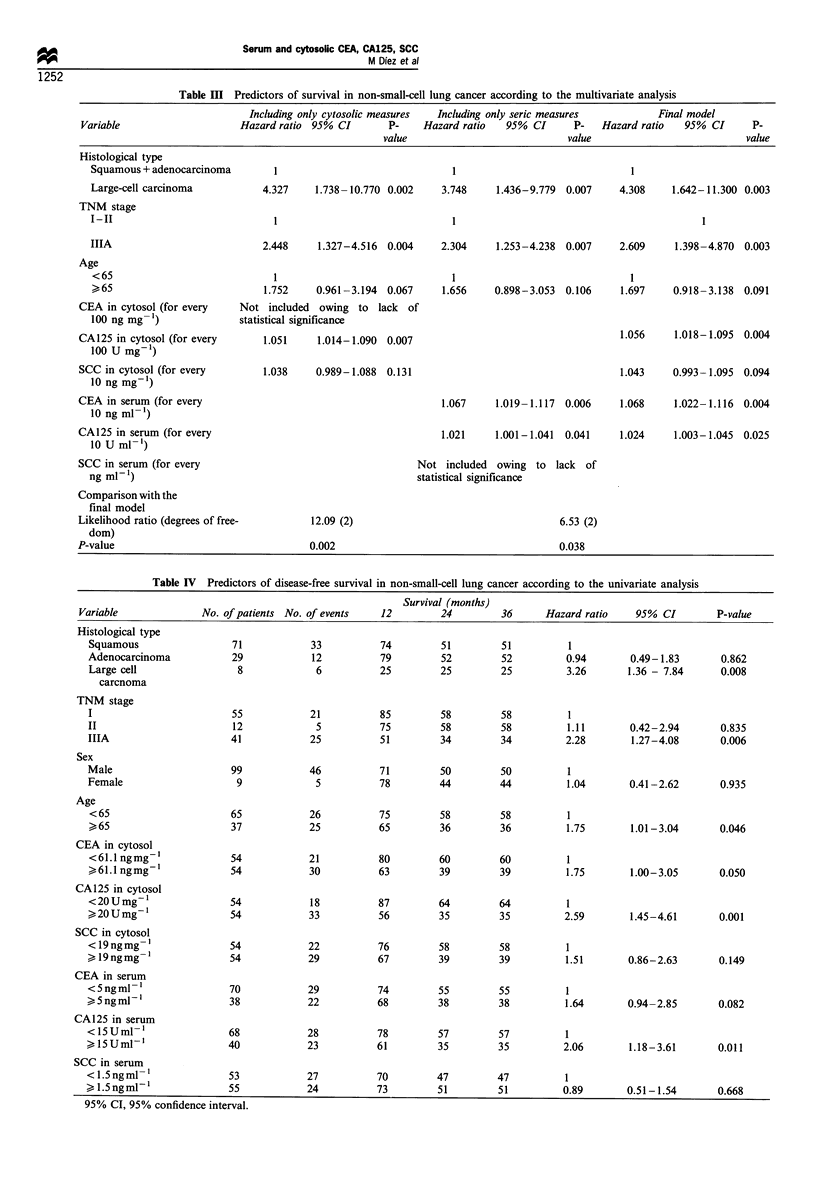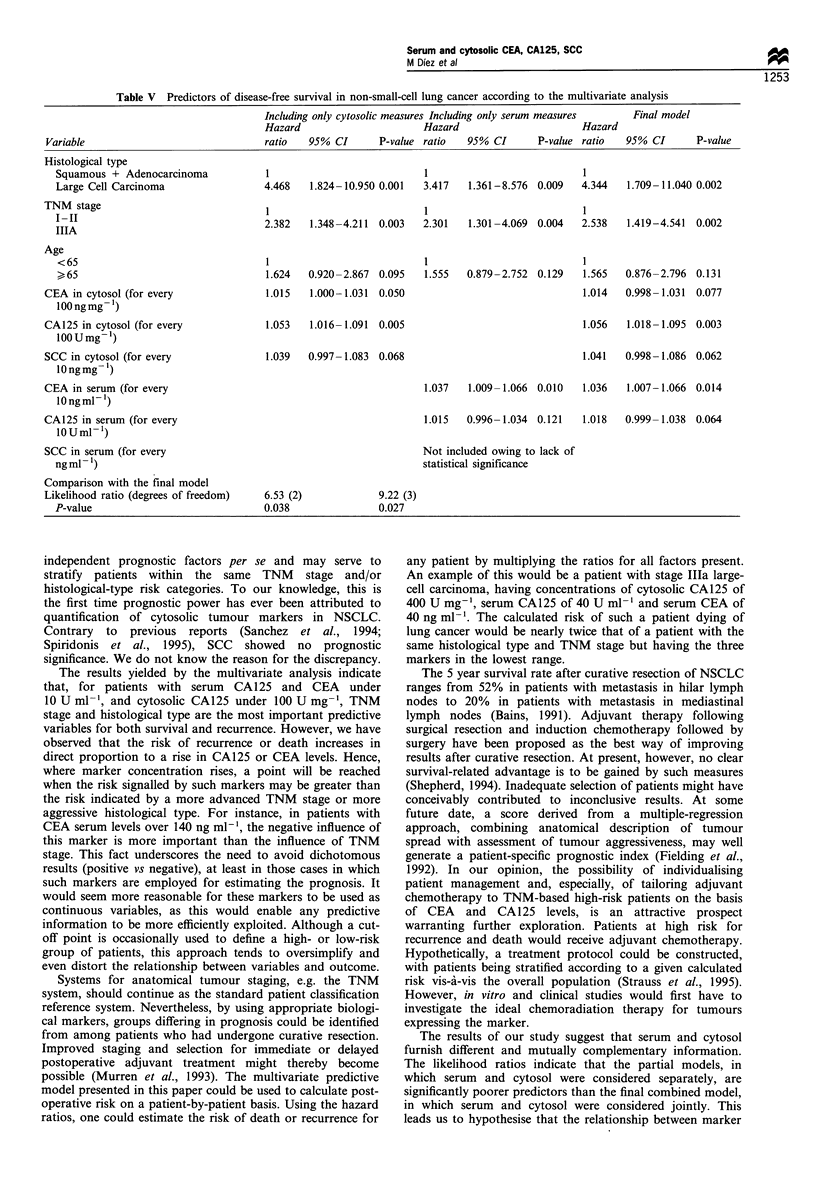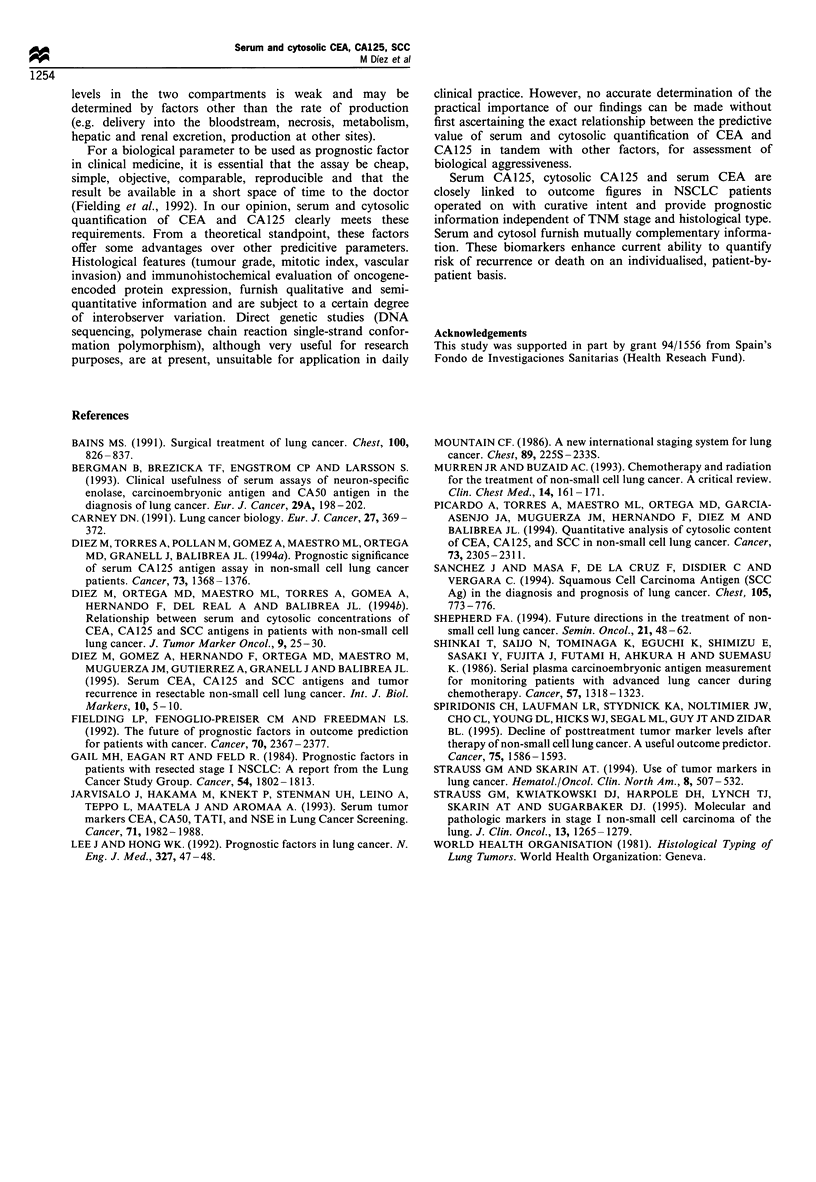# Prediction of survival and recurrence by serum and cytosolic levels of CEA, CA125 and SCC antigens in resectable non-small-cell lung cancer.

**DOI:** 10.1038/bjc.1996.239

**Published:** 1996-05

**Authors:** M. Díez, A. Torres, M. L. Maestro, M. D. Ortega, A. Gómez, M. Pollán, J. A. Lopez, A. Picardo, F. Hernando, J. L. Balibrea

**Affiliations:** San Carlos University Hospital, Madrid, Spain.

## Abstract

Risk of death and risk of recurrence in 108 potentially curable non-small-cell lung cancer patients were analysed with respect of TNM stage, histological type and carcinoembryonic antigen (CEA), CA125 antigen and squamous cell carcinoma antigen (SCC) levels in serum and cytosol. CA125 and CEA levels were closely related to outcome figures. Multivariate analyses indicated that TNM stage and histological type had the best predictive power, but serum and cytosolic CA125 and serum CEA contained additional, independent prognostic information. Predictive information drawn from serum and cytosolic levels proved mutually complementary. We conclude that CA125 and CEA complement TNM classification and histological type for the purpose of quantifying risk of death or recurrence.


British Journal of Cancer (1996) 73, 1248-1254
© 1996 Stockton Press All rights reserved 0007-0920/96 $12.00

### Prediction of survival and recurrence by serum and cytosolic levels of CEA, CA125 and SCC antigens in resectable non-small-cell lung cancer

M Díez[1]*, A Torres[1], ML Maestro[1], MD Ortega[1], A Gómez[1], M Pollán[2], JA Lopez[1], A Picardo[1], F Hernando[1] and JL Balibrea[1]

[1]*General Surgery II, San Carlos University Hospital, Madrid, Spain;* [2]*Cancer Epidemiology Unit, National Centre of Epidemiology, Instituto de Salud 'Carlos III', Madrid, Spain.*

**Summary** Risk of death and risk of recurrence in 108 potentially curable non-small-cell lung cancer patients were analysed with respect to TNM stage, histological type and carcinoembryonic antigen (CEA), CA125 antigen and squamous cell carcinoma antigen (SCC) levels in serum and cytosol. CA125 and CEA levels were closely related to outcome figures. Multivariate analyses indicated that TNM stage and histological type had the best predictive power, but serum and cytosolic CA125 and serum CEA contained additional, independent prognostic information. Predictive information drawn from serum and cytosolic levels proved mutually complementary. We conclude that CA125 and CEA complement TNM classification and histological type for the purpose of quantifying risk of death or recurrence.

**Keywords:** carcinoembryonic antigen; CA125; squamous cell carcinoma antigen; lung cancer; prognostic factor; survival

The tumour–node–metastasis (TNM) classification system is the cornerstone for planning therapy options in patients with non-small-cell lung cancer (NSCLC) (Bains, 1991). Such staging of tumour spread is the best available prognostic factor. However, its predictive power is limited. Differences may be seen between expected and actual outcome after curative resection among patients within the same TNM category of risk. Efforts are under way to confirm the possibility that long-term results or response to treatment may be partially based on biological characteristics inherent in tumour cells (Carney, 1991). Methods for the description of biological tumour aggressiveness are rapidly expanding (Carney, 1991; Lee and Hong, 1992).

Owing to their low sensitivity and specificity, tumour markers have, until now, played a less important role in the diagnosis and management of NSCLC than has been the case with most other common cancers (Bergman *et al.*, 1993; Strauss and Skarin, 1994; Jarvisalo *et al.*, 1993). Nevertheless, tumour markers might have a wide range of potential applications within the field of NSCLC as tools for description of tumour biological aggressiveness. Of these tumour markers, carcinoembryonic antigen (CEA) and squamous cell carcinoma antigen (SCC) have been two of the most commonly used to date. Their sensitivity for detecting the primary tumour is low, ranging from 30% to 60% (Strauss and Skarin, 1994) depending upon histological type and TNM stage. Several authors have reported that serum CEA and serum SCC assay provide useful information for establishing preoperative prognosis in patients with localised and resectable disease (Gail *et al.*, 1984; Sanchez *et al.*, 1994), guiding follow-up after surgical treatment (Díez *et al.*, 1995) and monitoring response to chemotherapy in advanced disease (Shinkai *et al.*, 1986; Spiridonis *et al.*, 1995). CA125 was initially described as an ovarian cancer-associated antigen, and has recently been assayed in NSCLC. In a previous study we reported that serum levels of CA125 provide independent information on survival and tumour relapse in patients undergoing curative surgical treatment for NSCLC (Díez *et al.*, 1994*a*).

Correspondence: M Díez, Cirugía General, Hospital Príncipe de Asturias, Universidad de Alcalá de Henares, 28805 Madrid, Spain.
*Present address: General Surgery, Príncipe de Asturias Hospital, University of Alcalá de Henares, 28805 Madrid, Spain.
Received 17 July 1995; revised 24 November 1995; accepted 14 December 1995

Analysis of tumour marker expression in NSCLC tumour tissue has not been reported as frequently. In a previous study we observed that cytosolic concentration of CEA, SCC and CA125 is a particular and distinctive characteristic of each histological type, something which could aid pathological classification (Picardo *et al.*, 1994). In addition, high CEA plus high CA125 content allows for identification of the large-cell carcinoma histological subtype (Picardo *et al.*, 1994). In our opinion, this kind of study could lead to a better understanding of the relationship between tumour marker and the biological features of the neoplasm.

This study aimed at assessing the ability of preoperative serum and cytosolic levels of CEA, CA125 and SCC antigens to provide information on the risk of death and recurrence in patients who had undergone curative surgical treatment for NSCLC.

### Patients and methods

### *Population*

A total of 108 histologically proven NSCLC patients (99 men and nine women; mean age 61 years) (s.d. 9 years) who underwent curative resection of the tumour between October 1989 and October 1993 were included in the study. They were consecutively submitted to our unit for surgical treatment. Patients undergoing chemotherapy or radiation therapy before surgery were not included. Careful, complete sampling of mediastinal lymph node groups was performed routinely before resection of the primary lesion. Patients who died of complications after surgery during this period (operative death) were not included. Histopathological diagnosis was carried out in accordance with the WHO classification of lung tumours (World Health Organization, 1981): 71 patients (65.7%) had squamous carcinoma, 29 (27%) adenocarcinoma and eight (7.3%), large-cell carcinoma. TNM staging (Mountain, 1986) was performed by correlating the operative and histological findings: 55 patients (51%) were in stage I, 12 (11%) in stage II and 41 (38%) in IIIA. Follow-up was conducted prospectively. During this period, two patients died as a result of unrelated causes, 3 and 16 months after surgery respectively. Two patients were lost to follow-up. Tumour recurrence was diagnosed in 51 patients, 45 of whom have since died. Median survival time of patients still living stands at 25 months (range 10–55).

*Specimens analysed and tumour marker assay*

Serum and lung samples were obtained from all these patients. Serum was obtained preoperatively. One sample from the lung tumour was always obtained at the time of surgery. The excised lung specimens were divided, one piece being sent for histological examination and the other for tumour marker assay. This specimen was cleaned of any necrotic tissue, washed with ice-cold saline and immediately frozen in liquid nitrogen. Total time elapsed from removal of specimen to freezing was less than 15 min. Cytosols were analysed immediately or stored at −80°C until assayed. Separation and processing of cytosols was effected by a previously described method (Picardo *et al.*, 1994). Commercially available kits were used and applied according to the manufacturer's instructions: enzyme immunoassay for CEA and CA125 (Hoffmann LaRoche, Basle, Switzerland), radioimmunoassay for SCC (Abbott Laboratories, Wiesbaden-Delkenheim, Germany). Cytosol protein concentration was determined by the Lowry method. Results of CA125 in cytosol are shown as U $mg^{-1}$ of proteins. Results of CEA and SCC in cytosol are shown as ng $mg^{-1}$ of proteins.

Performance characteristics of immunometric assays depend upon the matrix of the sample. Commercially available kits are standardised for serum. As a prior step, we therefore had to validate the technique for the cytosol matrix. In order to ascertain the precision and accuracy of the assay, the first ten lung cancer samples were divided into four parts. All the steps of the analysis were repeated in these

**Table I** Distribution of the three markers in cytosol and serum

| | *Markers in cytosol* | *Markers in serum* |
|---|---|---|
| CEA | | |
| Mean (s.d.) | 417.0 (1762.7) | 26.5 (97.2) |
| Median (ID) | 61.1 (14.0–140.0) | 3.1 (2.2–8.0) |
| Range | 0.1–4730 | 0.1–874 |
| CA125 | | |
| Mean (s.d.) | 136.5 (596.4) | 25.5 (95.2) |
| Median (ID) | 20.0 (4.8–69.0) | 9.5 (2.2–20.0) |
| Range | 0.1–6006 | 0.1–968 |
| SCC | | |
| Mean (s.d.) | 44.4 (61.2) | 3.2 (6.4) |
| Median (ID) | 19.0 (4.0–55.0) | 1.6 (0.5–3.4) |
| Range | 0.1–346 | 0.0–48 |

s.d., standard deviation. ID, interquartile distance.

**Table II** Predictors of survival in non-small cell lung cancer according to the univariate analysis.

| *Variable* | *No. of patients* | *No. of events* | *Survival (months)* 12 | 24 | 36 | *Hazard ratio* | *95%CI* | P-*value* |
|---|---|---|---|---|---|---|---|---|
| Histological type | | | | | | | | |
| Squamous | 71 | 29 | 87 | 66 | 44 | 1 | | |
| Adenocarcinoma | 29 | 10 | 86 | 66 | 54 | 0.84 | 0.41–1.73 | 0.642 |
| Large-cell carcinoma | 8 | 6 | 38 | 25 | 25 | 3.44 | 1.41–8.38 | 0.007 |
| TNM stage | | | | | | | | |
| I | 55 | 18 | 94 | 73 | 51 | 1 | | |
| II | 12 | 5 | 83 | 75 | 54 | 1.14 | 0.42–3.07 | 0.799 |
| IIIA | 41 | 22 | 67 | 43 | 33 | 2.41 | 1.29–4.50 | 0.006 |
| Sex | | | | | | | | |
| Male | 99 | 40 | 82 | 65 | 45 | 1 | | |
| Female | 9 | 5 | 100 | 44 | 44 | 1.21 | 0.48–3.06 | 0.695 |
| Age | | | | | | | | |
| <65 | 65 | 23 | 88 | 72 | 53 | 1 | | |
| ⩾65 | 37 | 22 | 75 | 47 | 34 | 1.72 | 0.96–3.09 | 0.069 |
| CEA in cytosol | | | | | | | | |
| <61.1 ng $mg^{-1}$ | 54 | 19 | 89 | 70 | 49 | 1 | | |
| ⩾61.1 ng $mg^{-1}$ | 54 | 26 | 77 | 56 | 42 | 1.52 | 0.84–2.75 | 0.167 |
| CA125 in cytosol | | | | | | | | |
| <20 U $mg^{-1}$ | 54 | 14 | 96 | 79 | 60 | 1 | | |
| ⩾20 U $mg^{-1}$ | 54 | 31 | 69 | 46 | 32 | 2.92 | 1.55–5.50 | <0.001 |
| SCC in cytosol | | | | | | | | |
| <19 ng $mg^{-1}$ | 54 | 19 | 85 | 67 | 55 | 1 | | |
| ⩾19 ng $mg^{-1}$ | 54 | 26 | 81 | 57 | 35 | 1.54 | 0.85–2.78 | 0.155 |
| CEA in serum | | | | | | | | |
| <5 ng $ml^{-1}$ | 70 | 26 | 85 | 63 | 53 | 1 | | |
| ⩾5 ng $ml^{-1}$ | 38 | 19 | 78 | 62 | 33 | 1.45 | 0.80–2.61 | 0.221 |
| CA125 in serum | | | | | | | | |
| <15 U $ml^{-1}$ | 68 | 25 | 88 | 68 | 54 | 1 | | |
| ⩾15 U $ml^{-1}$ | 40 | 20 | 74 | 53 | 29 | 1.92 | 1.06–3.47 | 0.031 |
| SCC in serum | | | | | | | | |
| <1.5 ng $ml^{-1}$ | 53 | 25 | 83 | 62 | 40 | 1 | | |
| ⩾1.5 ng $ml^{-1}$ | 55 | 20 | 83 | 63 | 51 | 0.79 | 0.44–1.42 | 0.427 |

95% CI, 95% confidence interval.

**Figure 1** Cumulative probability of survival by TNM stages and cytosolic concentration of CA125. Patients with tumour marker level lower than the cut-off (——), patients with levels higher than the cut-off (- - - -). (a) Comparison in stages I–II: log-rank $P=0.013$. (b) Comparison in stage IIIa: log-rank $P=0.020$.

**Figure 2** Cumulative probability of survival in patients with squamous carcinoma and adenocarcinoma categorised by cytosolic concentration of CA125. Patients with tumour marker level lower than the cut-off (——), patients with levels higher than the cut-off (- - - -). (a) Comparison in squamous carcinoma: log-rank $P<0.001$. (b) Comparison in adenocarcinoma: log-rank $P=0.233$.

specimens separately. Variation coefficients of the technique for cytosols were 15% for CEA, 13% for CA125 and 16% for SCC. Intra-assay variation coefficients were 7% in the CEA assay, 6% in CA125 and 8% in SCC. Interassay variation coefficients were 10% in the CEA assay, 9% in CA125 and 11% in SCC.

### *Statistical analysis*

In the statistical analysis, median and interquartile distances were used as summary measures, owing to the asymmetric distribution of marker values. Survival and recurrence rates linked to tumour marker levels were studied using the Kaplan–Meier method, and differences between subgroups of patients were compared using Mantel's log-rank test. Survival and disease-free survival were calculated from surgery to last contact or death and from surgery to recurrence respectively. Patients lost to follow-up and deaths due to a different cause were considered as censored. The importance of all prognostic factors considered for both survival and recurrence was estimated using Cox's proportional hazards regression model. The crude effect of each predictor was evaluated, using unadjusted hazard ratios as an estimate of relative risk. Possible interactions between tumour markers and the other predictors were tested. In the univariate study of survival, the tumour markers were analysed as dichotomous variables. The following cut-offs were used for serum: CEA, 5 ng $ml^{-1}$; CA125, 15 U $ml^{-1}$;

SCC, 1.5 ng $ml^{-1}$. These values had been selected in a previous study by our group by studying the receiver operating characteristic curves (Díez *et al*., 1994*a*,*b*). No generally accepted values exist as cut-offs for cytosol, and we therefore decided to use the median value. In the absence of any other reference, this has the advantage of furnishing a balanced distribution of the sample. Accordingly, the cut-offs for cytosol were: CEA, 61.1 ng $mg^{-1}$; CA125, 20 U $mg^{-1}$; SCC, 19 ng $mg^{-1}$. Sample size was not pre-established, but was mainly determined by population incidence and thus by the number of patients attending our hospital.

**Figure 3** Cumulative probability of survival in patients with squamous carcinoma and adenocarcinoma categorised by serum concentration of CEA. Patients with tumour marker level lower than the cut-off (——), patients with levels higher than the cut-off (- - - -). (a) Comparison in squamous carcinoma: log-rank $P=0.91$. (b) Comparison in adenocarcinoma: log-rank $P=0.04$.

### Results

Distribution of serum and cytosolic concentration of CEA, CA125 and SCC are depicted in Table I. Concentrations of all three markers were far higher in cytosol than in serum; likewise, the degree of dispersion was comparatively greater in the cytosol-based results.

### *Survival*

The cumulative probability of survival over 36 month follow-up was 45%. In the univariate analysis this result was significantly related to histological type, TNM stage and CA125 levels in serum and cytosol (Table II). Patients with large cell carcinoma showed significantly lower probability of survival than patients with squamous carcinoma and adenocarcinoma ($P=0.007$), but no significant differences were detected between the latter two histological types ($P=0.642$). Whereas stage IIIa patients showed significantly lower probability of survival than patients in stages I and II ($P=0.006$), no differences were detected between the latter two ($P=0.799$). High CA125 cytosolic levels were associated with a significantly lower probability of survival (32% *vs* 60%) ($P<0.001$), as were high CA125 serum levels (29% *vs* 54%) ($P=0.031$). When survival estimates for histological type and TNM stage were classified according to results of CA125 in cytosol, patients with high levels of the marker exhibited a significantly worse outcome (Figures 1 and 2). Likewise, within adenocarcinoma, patients with high serum CEA levels exhibited significantly lower survival than those with low serum CEA (Figure 3).

Table III sets out the results of the multivariate analysis. Shown in this table are: a first model, including the results of cytosolic measurements after adjustment for histological type, TNM stage and age; a second model, including serum measurements after adjustment for the same three factors; and a third model, including only those variables showing significant influence in the first two models. Interaction terms did not significantly improve any of these models. The likelihood ratios indicate that the partial models were significantly poorer predictors than the final combined model. In this final model, TNM stage and histological type proved to be the most important predictors of survival. Cytosolic and serum levels of CA125, and serum CEA, once adjusted for other prognostic variables, revealed themselves to be independent predictive factors. Table III shows that the risk of death increases in direct proportion to a rise in cytosolic CA125, serum CA125 and serum CEA.

### *Recurrence*

The 36 month disease-free survival rate was 47%. In the univariate analysis, this result was related to histological type, TNM stage, age, cytosolic CEA and serum and cytosolic CA125 (Table IV). High CA125 cytosolic and CEA serum levels were associated with a significantly lower probability of disease-free survival.

Table V sets out the results of the multivariate analysis. In the final model, TNM stage and histological type again proved to be the most important predictors. Cytosolic CA125 and serum CEA revealed themselves to be independent predictive factors. Table V illustrates that risk of recurrence increases in direct proportion to a rise in cytosolic CA125 and serum CEA.

### Discussion

Preoperative serum CA125, cytosolic CA125 and preoperative serum CEA were observed to be closely related to outcome figures in NSCLC. These markers provided prognostic information not taken into account by TNM stage or histological type. The data indicate that CA125 and CEA levels do not simply reflect tumour load but are

**Table III** Predictors of survival in non-small-cell lung cancer according to the multivariate analysis

| *Variable* | *Including only cytosolic measures* | | | *Including only seric measures* | | | *Final model* | | |
|---|---|---|---|---|---|---|---|---|---|
| | *Hazard ratio* | *95% CI* | P-*value* | *Hazard ratio* | *95% CI* | P-*value* | *Hazard ratio* | *95% CI* | P-*value* |
| Histological type | | | | | | | | | |
| Squamous + adenocarcinoma | 1 | | | 1 | | | 1 | | |
| Large-cell carcinoma | 4.327 | 1.738–10.770 | 0.002 | 3.748 | 1.436–9.779 | 0.007 | 4.308 | 1.642–11.300 | 0.003 |
| TNM stage | | | | | | | | | |
| I–II | 1 | | | 1 | | | 1 | | |
| IIIA | 2.448 | 1.327–4.516 | 0.004 | 2.304 | 1.253–4.238 | 0.007 | 2.609 | 1.398–4.870 | 0.003 |
| Age | | | | | | | | | |
| <65 | 1 | | | 1 | | | 1 | | |
| ⩾65 | 1.752 | 0.961–3.194 | 0.067 | 1.656 | 0.898–3.053 | 0.106 | 1.697 | 0.918–3.138 | 0.091 |
| CEA in cytosol (for every 100 ng $mg^{-1}$) | Not included owing to lack of statistical significance | | | | | | | | |
| CA125 in cytosol (for every 100 U $mg^{-1}$) | 1.051 | 1.014–1.090 | 0.007 | | | | 1.056 | 1.018–1.095 | 0.004 |
| SCC in cytosol (for every 10 ng $mg^{-1}$) | 1.038 | 0.989–1.088 | 0.131 | | | | 1.043 | 0.993–1.095 | 0.094 |
| CEA in serum (for every 10 ng $ml^{-1}$) | | | | 1.067 | 1.019–1.117 | 0.006 | 1.068 | 1.022–1.116 | 0.004 |
| CA125 in serum (for every 10 U $ml^{-1}$) | | | | 1.021 | 1.001–1.041 | 0.041 | 1.024 | 1.003–1.045 | 0.025 |
| SCC in serum (for every ng $ml^{-1}$) | | | | Not included owing to lack of statistical significance | | | | | |
| Comparison with the final model | | | | | | | | | |
| Likelihood ratio (degrees of freedom) | | 12.09 (2) | | | | 6.53 (2) | | | |
| *P*-value | | 0.002 | | | | 0.038 | | | |

**Table IV** Predictors of disease-free survival in non-small-cell lung cancer according to the univariate analysis

| *Variable* | *No. of patients* | *No. of events* | *Survival (months)* 12 | 24 | 36 | *Hazard ratio* | *95% CI* | P-*value* |
|---|---|---|---|---|---|---|---|---|
| Histological type | | | | | | | | |
| Squamous | 71 | 33 | 74 | 51 | 51 | 1 | | |
| Adenocarcinoma | 29 | 12 | 79 | 52 | 52 | 0.94 | 0.49–1.83 | 0.862 |
| Large cell carcnoma | 8 | 6 | 25 | 25 | 25 | 3.26 | 1.36 – 7.84 | 0.008 |
| TNM stage | | | | | | | | |
| I | 55 | 21 | 85 | 58 | 58 | 1 | | |
| II | 12 | 5 | 75 | 58 | 58 | 1.11 | 0.42–2.94 | 0.835 |
| IIIA | 41 | 25 | 51 | 34 | 34 | 2.28 | 1.27–4.08 | 0.006 |
| Sex | | | | | | | | |
| Male | 99 | 46 | 71 | 50 | 50 | 1 | | |
| Female | 9 | 5 | 78 | 44 | 44 | 1.04 | 0.41–2.62 | 0.935 |
| Age | | | | | | | | |
| <65 | 65 | 26 | 75 | 58 | 58 | 1 | | |
| ⩾65 | 37 | 25 | 65 | 36 | 36 | 1.75 | 1.01–3.04 | 0.046 |
| CEA in cytosol | | | | | | | | |
| <61.1 ng $mg^{-1}$ | 54 | 21 | 80 | 60 | 60 | 1 | | |
| ⩾61.1 ng $mg^{-1}$ | 54 | 30 | 63 | 39 | 39 | 1.75 | 1.00–3.05 | 0.050 |
| CA125 in cytosol | | | | | | | | |
| <20 U $mg^{-1}$ | 54 | 18 | 87 | 64 | 64 | 1 | | |
| ⩾20 U $mg^{-1}$ | 54 | 33 | 56 | 35 | 35 | 2.59 | 1.45–4.61 | 0.001 |
| SCC in cytosol | | | | | | | | |
| <19 ng $mg^{-1}$ | 54 | 22 | 76 | 58 | 58 | 1 | | |
| ⩾19 ng $mg^{-1}$ | 54 | 29 | 67 | 39 | 39 | 1.51 | 0.86–2.63 | 0.149 |
| CEA in serum | | | | | | | | |
| <5 ng $ml^{-1}$ | 70 | 29 | 74 | 55 | 55 | 1 | | |
| ⩾5 ng $ml^{-1}$ | 38 | 22 | 68 | 38 | 38 | 1.64 | 0.94–2.85 | 0.082 |
| CA125 in serum | | | | | | | | |
| <15 U $ml^{-1}$ | 68 | 28 | 78 | 57 | 57 | 1 | | |
| ⩾15 U $ml^{-1}$ | 40 | 23 | 61 | 35 | 35 | 2.06 | 1.18–3.61 | 0.011 |
| SCC in serum | | | | | | | | |
| <1.5 ng $ml^{-1}$ | 53 | 27 | 70 | 47 | 47 | 1 | | |
| ⩾1.5 ng $ml^{-1}$ | 55 | 24 | 73 | 51 | 51 | 0.89 | 0.51–1.54 | 0.668 |

95% CI, 95% confidence interval.

Table V Predictors of disease-free survival in non-small-cell lung cancer according to the multivariate analysis

| Variable | *Including only cytosolic measures* Hazard ratio | 95% CI | P-value | *Including only serum measures* Hazard ratio | 95% CI | P-value | *Final model* Hazard ratio | 95% CI | P-value |
|---|---|---|---|---|---|---|---|---|---|
| Histological type | | | | | | | | | |
| Squamous + Adenocarcinoma | 1 | | | 1 | | | 1 | | |
| Large Cell Carcinoma | 4.468 | 1.824–10.950 | 0.001 | 3.417 | 1.361–8.576 | 0.009 | 4.344 | 1.709–11.040 | 0.002 |
| TNM stage | | | | | | | | | |
| I–II | 1 | | | 1 | | | 1 | | |
| IIIA | 2.382 | 1.348–4.211 | 0.003 | 2.301 | 1.301–4.069 | 0.004 | 2.538 | 1.419–4.541 | 0.002 |
| Age | | | | | | | | | |
| <65 | 1 | | | 1 | | | 1 | | |
| ⩾65 | 1.624 | 0.920–2.867 | 0.095 | 1.555 | 0.879–2.752 | 0.129 | 1.565 | 0.876–2.796 | 0.131 |
| CEA in cytosol (for every 100 ng mg$^{-1}$) | 1.015 | 1.000–1.031 | 0.050 | | | | 1.014 | 0.998–1.031 | 0.077 |
| CA125 in cytosol (for every 100 U mg$^{-1}$) | 1.053 | 1.016–1.091 | 0.005 | | | | 1.056 | 1.018–1.095 | 0.003 |
| SCC in cytosol (for every 10 ng mg$^{-1}$) | 1.039 | 0.997–1.083 | 0.068 | | | | 1.041 | 0.998–1.086 | 0.062 |
| CEA in serum (for every 10 ng ml$^{-1}$) | | | | 1.037 | 1.009–1.066 | 0.010 | 1.036 | 1.007–1.066 | 0.014 |
| CA125 in serum (for every 10 U ml$^{-1}$) | | | | 1.015 | 0.996–1.034 | 0.121 | 1.018 | 0.999–1.038 | 0.064 |
| SCC in serum (for every ng ml$^{-1}$) | | | | Not included owing to lack of statistical significance | | | | | |
| Comparison with the final model | | | | | | | | | |
| Likelihood ratio (degrees of freedom) | 6.53 (2) | | 9.22 (3) | | | | | | |
| *P*-value | 0.038 | | 0.027 | | | | | | |

independent prognostic factors *per se* and may serve to stratify patients within the same TNM stage and/or histological-type risk categories. To our knowledge, this is the first time prognostic power has ever been attributed to quantification of cytosolic tumour markers in NSCLC. Contrary to previous reports (Sanchez *et al.*, 1994; Spiridonis *et al.*, 1995), SCC showed no prognostic significance. We do not know the reason for the discrepancy.

The results yielded by the multivariate analysis indicate that, for patients with serum CA125 and CEA under 10 U ml$^{-1}$, and cytosolic CA125 under 100 U mg$^{-1}$, TNM stage and histological type are the most important predictive variables for both survival and recurrence. However, we have observed that the risk of recurrence or death increases in direct proportion to a rise in CA125 or CEA levels. Hence, where marker concentration rises, a point will be reached when the risk signalled by such markers may be greater than the risk indicated by a more advanced TNM stage or more aggressive histological type. For instance, in patients with CEA serum levels over 140 ng ml$^{-1}$, the negative influence of this marker is more important than the influence of TNM stage. This fact underscores the need to avoid dichotomous results (positive *vs* negative), at least in those cases in which such markers are employed for estimating the prognosis. It would seem more reasonable for these markers to be used as continuous variables, as this would enable any predictive information to be more efficiently exploited. Although a cut-off point is occasionally used to define a high- or low-risk group of patients, this approach tends to oversimplify and even distort the relationship between variables and outcome.

Systems for anatomical tumour staging, e.g. the TNM system, should continue as the standard patient classification reference system. Nevertheless, by using appropriate biological markers, groups differing in prognosis could be identified from among patients who had undergone curative resection. Improved staging and selection for immediate or delayed postoperative adjuvant treatment might thereby become possible (Murren *et al.*, 1993). The multivariate predictive model presented in this paper could be used to calculate post-operative risk on a patient-by-patient basis. Using the hazard ratios, one could estimate the risk of death or recurrence for any patient by multiplying the ratios for all factors present. An example of this would be a patient with stage IIIa large-cell carcinoma, having concentrations of cytosolic CA125 of 400 U mg$^{-1}$, serum CA125 of 40 U ml$^{-1}$ and serum CEA of 40 ng ml$^{-1}$. The calculated risk of such a patient dying of lung cancer would be nearly twice that of a patient with the same histological type and TNM stage but having the three markers in the lowest range.

The 5 year survival rate after curative resection of NSCLC ranges from 52% in patients with metastasis in hilar lymph nodes to 20% in patients with metastasis in mediastinal lymph nodes (Bains, 1991). Adjuvant therapy following surgical resection and induction chemotherapy followed by surgery have been proposed as the best way of improving results after curative resection. At present, however, no clear survival-related advantage is to be gained by such measures (Shepherd, 1994). Inadequate selection of patients might have conceivably contributed to inconclusive results. At some future date, a score derived from a multiple-regression approach, combining anatomical description of tumour spread with assessment of tumour aggressiveness, may well generate a patient-specific prognostic index (Fielding *et al.*, 1992). In our opinion, the possibility of individualising patient management and, especially, of tailoring adjuvant chemotherapy to TNM-based high-risk patients on the basis of CEA and CA125 levels, is an attractive prospect warranting further exploration. Patients at high risk for recurrence and death would receive adjuvant chemotherapy. Hypothetically, a treatment protocol could be constructed, with patients being stratified according to a given calculated risk vis-à-vis the overall population (Strauss *et al.*, 1995). However, *in vitro* and clinical studies would first have to investigate the ideal chemoradiation therapy for tumours expressing the marker.

The results of our study suggest that serum and cytosol furnish different and mutually complementary information. The likelihood ratios indicate that the partial models, in which serum and cytosol were considered separately, are significantly poorer predictors than the final combined model, in which serum and cytosol were considered jointly. This leads us to hypothesise that the relationship between marker levels in the two compartments is weak and may be determined by factors other than the rate of production (e.g. delivery into the bloodstream, necrosis, metabolism, hepatic and renal excretion, production at other sites).

For a biological parameter to be used as prognostic factor in clinical medicine, it is essential that the assay be cheap, simple, objective, comparable, reproducible and that the result be available in a short space of time to the doctor (Fielding *et al.*, 1992). In our opinion, serum and cytosolic quantification of CEA and CA125 clearly meets these requirements. From a theoretical standpoint, these factors offer some advantages over other predicitive parameters. Histological features (tumour grade, mitotic index, vascular invasion) and immunohistochemical evaluation of oncogene-encoded protein expression, furnish qualitative and semi-quantitative information and are subject to a certain degree of interobserver variation. Direct genetic studies (DNA sequencing, polymerase chain reaction single-strand conformation polymorphism), although very useful for research purposes, are at present, unsuitable for application in daily clinical practice. However, no accurate determination of the practical importance of our findings can be made without first ascertaining the exact relationship between the predictive value of serum and cytosolic quantification of CEA and CA125 in tandem with other factors, for assessment of biological aggressiveness.

Serum CA125, cytosolic CA125 and serum CEA are closely linked to outcome figures in NSCLC patients operated on with curative intent and provide prognostic information independent of TNM stage and histological type. Serum and cytosol furnish mutually complementary information. These biomarkers enhance current ability to quantify risk of recurrence or death on an individualised, patient-by-patient basis.

**Acknowledgements**
This study was supported in part by grant 94/1556 from Spain's Fondo de Investigaciones Sanitarias (Health Reseach Fund).

### References

BAINS MS. (1991). Surgical treatment of lung cancer. *Chest*, **100,** 826–837.

BERGMAN B, BREZICKA TF, ENGSTROM CP AND LARSSON S. (1993). Clinical usefulness of serum assays of neuron-specific enolase, carcinoembryonic antigen and CA50 antigen in the diagnosis of lung cancer. *Eur. J. Cancer*, **29A**, 198–202.

CARNEY DN. (1991). Lung cancer biology. *Eur. J. Cancer*, **27,** 369–372.

DIEZ M, TORRES A, POLLAN M, GOMEZ A, MAESTRO ML, ORTEGA MD, GRANELL J, BALIBREA JL. (1994*a*). Prognostic significance of serum CA125 antigen assay in non-small cell lung cancer patients. *Cancer*, **73,** 1368–1376.

DIEZ M, ORTEGA MD, MAESTRO ML, TORRES A, GOMEA A, HERNANDO F, DEL REAL A AND BALIBREA JL. (1994*b*). Relationship between serum and cytosolic concentrations of CEA, CA125 and SCC antigens in patients with non-small cell lung cancer. *J. Tumor Marker Oncol.*, **9,** 25–30.

DIEZ M, GOMEZ A, HERNANDO F, ORTEGA MD, MAESTRO M, MUGUERZA JM, GUTIERREZ A, GRANELL J AND BALIBREA JL. (1995). Serum CEA, CA125 and SCC antigens and tumor recurrence in resectable non-small cell lung cancer. *Int. J. Biol. Markers*, **10,** 5–10.

FIELDING LP, FENOGLIO-PREISER CM AND FREEDMAN LS. (1992). The future of prognostic factors in outcome prediction for patients with cancer. *Cancer*, **70,** 2367–2377.

GAIL MH, EAGAN RT AND FELD R. (1984). Prognostic factors in patients with resected stage I NSCLC: A report from the Lung Cancer Study Group. *Cancer*, **54,** 1802–1813.

JARVISALO J, HAKAMA M, KNEKT P, STENMAN UH, LEINO A, TEPPO L, MAATELA J AND AROMAA A. (1993). Serum tumor markers CEA, CA50, TATI, and NSE in Lung Cancer Screening. *Cancer*, **71,** 1982–1988.

LEE J AND HONG WK. (1992). Prognostic factors in lung cancer. *N. Eng. J. Med.*, **327,** 47–48.

MOUNTAIN CF. (1986). A new international staging system for lung cancer. *Chest*, **89,** 225S–233S.

MURREN JR AND BUZAID AC. (1993). Chemotherapy and radiation for the treatment of non-small cell lung cancer. A critical review. *Clin. Chest Med.*, **14,** 161–171.

PICARDO A, TORRES A, MAESTRO ML, ORTEGA MD, GARCIA-ASENJO JA, MUGUERZA JM, HERNANDO F, DIEZ M AND BALIBREA JL. (1994). Quantitative analysis of cytosolic content of CEA, CA125, and SCC in non-small cell lung cancer. *Cancer*, **73,** 2305–2311.

SANCHEZ J AND MASA F, DE LA CRUZ F, DISDIER C AND VERGARA C. (1994). Squamous Cell Carcinoma Antigen (SCC Ag) in the diagnosis and prognosis of lung cancer. *Chest*, **105,** 773–776.

SHEPHERD FA. (1994). Future directions in the treatment of non-small cell lung cancer. *Semin. Oncol.*, **21,** 48–62.

SHINKAI T, SAIJO N, TOMINAGA K, EGUCHI K, SHIMIZU E, SASAKI Y, FUJITA J, FUTAMI H, AHKURA H AND SUEMASU K. (1986). Serial plasma carcinoembryonic antigen measurement for monitoring patients with advanced lung cancer during chemotherapy. *Cancer*, **57,** 1318–1323.

SPIRIDONIS CH, LAUFMAN LR, STYDNICK KA, NOLTIMIER JW, CHO CL, YOUNG DL, HICKS WJ, SEGAL ML, GUY JT AND ZIDAR BL. (1995). Decline of posttreatment tumor marker levels after therapy of non-small cell lung cancer. A useful outcome predictor. *Cancer*, **75,** 1586–1593.

STRAUSS GM AND SKARIN AT. (1994). Use of tumor markers in lung cancer. *Hematol./Oncol. Clin. North Am.*, **8,** 507–532.

STRAUSS GM, KWIATKOWSKI DJ, HARPOLE DH, LYNCH TJ, SKARIN AT AND SUGARBAKER DJ. (1995). Molecular and pathologic markers in stage I non-small cell carcinoma of the lung. *J. Clin. Oncol.*, **13,** 1265–1279.

WORLD HEALTH ORGANISATION (1981). *Histological Typing of Lung Tumors.* World Health Organization: Geneva.